# The Army Medical Department

**Published:** 1900-03-24

**Authors:** 


### The Hospital

A JOURNAL OF

The Medical Sciences and Hospital Administration.

VOL. XXVII.—No. 704.] EDITED BY SIR HENRY BURDETT, K.C.B., AND SOLOMON C. SMITH, M.D., M.R.C.P. [MARCH 24, 1900.

### THE ARMY MEDICAL DEPARTMENT.

DURING the discussion which took place in the House of Commons last Friday on the vote for the Army Medical Department, several points were raised which, although answered in a satisfactory spirit, cannot be regarded as entirely shelved by the pleasant words of Mr. Wyndham. Do not let us be thought extravagant. We quite agree that it would be impossible to keep up at all times an establishment of the Royal Army Medical Corps which would be adequate for a time when we are mobilising the whole of the army for an expedition. The experience of the war has shown that in every department, not even excepting the actual fighting line, place can usefully be found for "civilian" helpers, or, in more correct phraseology, for those who engage only for temporary service. Notably this must be the case in regard to the more purely medical and surgical work of the Royal Army Medical Corps, and more especially in regard to al that concerns the base hospitals, the proceedings in which most nearly resemble those of civil life. Nevertheless, the fact remains that much of the work of the Royal Army Medical Corps is of a highly specialised character, which cannot be taken up at a moment's notice by civilians; and, unfortunately, neither the Estimates nor the statement made to the House show much intention of removing what is one of the great causes of the unpopularity of the army medical service, namely, that chronic undermanning which has prevailed for many years.

Much has been done to render the army attractive to medical men, especially by the grant of military rank and title, and we take it that the two main things which deter men from applying for commissions are first and principally the undermanning of the corps, and secondly a certain lack of faith in Government promises, which is maintained by the persistence, in spite of all representations, of certain arrangements under which medical officers in the Imperial service are loaned to the Indian Government. The undermanning, however, with all that it involves, is the great thing. The difficulty of obtaining leave of any sort, even for sickness, not to mention leave for the purposes of study, and the resulting constant irritation which permeates the service is all the outcome of the undermanning of the department and the consequent over work of every individual in it. The position of an officer in the Royal Army Medical Corps is good, and while the work is professional and pleasant, it is entirely devoid of that galling appearance of "shopkeeping," which the taking of small fees from little people of necessity entails during the early years of general practice. There is very much in the career of the army medical officer which appeals to gentlemen who have received a medical education, ; there is in the medical profession a growing number of those who, being the sons of, and having been brought up in the society of, gentlemen, shrink from the semi-commercial fee-grubbing which so often characterises private practice ; and there seems no reason why a large and quite sufficient number of first-class medical men should not apply for commissions in the medical, for exactly the same reasons and influenced by exactly the same feelings as lead other men to enter the combatant branches, were it not for the undermanning of the department and the consequent difficulty about leave. Some people say the pay is too low, but we do not think that pay is everything. Everyone nowadays works not for money alone, and the man who cannot get his leave not only feels that he is "done," but feels also that he is declassed.

We are very much afraid that one result of all the praise that has been lavished upon the officers and men of the Royal Army Medical Corps will be to lead the War Office to think that this at least is a department which requires no reform. Our feeling would be just the opposite, namely, that a department which has done so well should at least have justice. Mr. Wyndham said to the House that they "could not pay too high a tribute to the fortitude, courage, and efficiency of the medical officers, which had been displayed." "They were not," he said, "exalted by the joy and intoxication of battle; they were sustained alone by their devotion to suffering humanity, and this rendered their services, in his opinion, all the more heroic." True ! And for this they ask no reward. They do but ask that their members shall be fitted for their work, and that provision shall be made for enabling them to keep themselves abreast of the advances of medical science. We are glad to believe with Mr. Wyndham that the medical schools are now furnishing a greater number of candidates, and we feel very sure that there are growing up in the medical schools a large number of good men who will gladly join if they can feel assured that the undermanning of the service which has characterised the department for so long, even in times of peace, will be done away with.

